# Examination of non-conventional dysplasias adjacent to colorectal adenocarcinoma in patients with IBD

**DOI:** 10.3389/pore.2024.1611978

**Published:** 2025-01-03

**Authors:** Szintia Almási, Zsófia Balajthy, Bence Baráth, Zsófia Krisztina Török, Panna Szaszák, Tamás Lantos, Bence Kővári, Anita Sejben

**Affiliations:** ^1^ Albert Szent-Györgyi Medical School, University of Szeged, Szeged, Hungary; ^2^ Mass General Brigham, Harvard Medical School, Boston, MA, United States

**Keywords:** non-conventional dysplasia, colorectal adenocarcinoma, inflammatory bowel disease, ulcerative colitis, Crohn’s disease

## Abstract

**Objective:**

Recently, several non-conventional variants of IBD-associated dysplasia have been described; however, their prevalence in Central-Eastern Europe is unknown. We aimed to perform a retrospective pilot study by re-evaluating several IBD-associated adenocarcinoma cases to survey the incidence of adjacent non-conventional dysplasia and validate that recent North American findings may apply to a European population.

**Methods:**

Retrospectively, 28 randomly chosen cases of IBD-associated adenocarcinomas diagnosed between 2010 and 2022 were re-evaluated. The patient’s sex, age (at the diagnosis of IBD and neoplasia), type of IBD, type of specimen [biopsy (n = 8)/surgical specimen (n = 20)], histological type, grade, localisation, stage, disease-free (DFS) and overall survival (OS) were obtained. Statistical analyses were carried out by using Mann-Whitney (continuous variables), Fisher’s exact (categorical variables), Kaplan-Meier (DFS/OS curves), and logrank test (survival curves).

**Results:**

Exclusively, conventional dysplasia was observed in 11, and non-conventional dysplasia in 8 patients. Combined conventional and non-conventional dysplasia was detected in 9 patients. Non-conventional dysplasia showing a combination of multiple subtypes was noted in 10 cases. Altogether, 25 non-conventional dysplastic foci were identified, which were diagnosed as hypermucinous (n = 9), goblet cell-deficient (n = 6), serrated not otherwise specified (NOS) (n = 6), and traditional serrated adenoma-like (n = 4). The majority of non-conventional dysplasias were associated with ulcerative colitis (n = 12). Mucinous adenocarcinoma was exclusively associated with non-conventional dysplasia, while medullary carcinoma was only with conventional dysplasias (*p = 0.014* and *0.041*).

**Conclusion:**

Based on our results, non-conventional dysplasia is common (60%) adjacent to IBD-associated adenocarcinomas in a Central-Eastern European population and may be detected in biopsies. As multiple recent publications reported evidence of a worse prognosis and more common flat morphology compared to conventional dysplasias, their recognition is of great importance, and stricter follow-up with random biopsy samples may be considered.

## Introduction

Among patients who live with inflammatory bowel disease (IBD), including Crohn’s disease (CD) and ulcerative colitis (UC), the risk of developing neoplasia is twofold compared to the normal population due to the chronic inflammatory milieu [1]. With effective anti-inflammatory treatment and surveillance, the risk of dysplasia or CRC has decreased in the last few decades [2, 3].

Besides conventional dysplasia and CRC, IBD patients are also at risk of developing non-conventional types of dysplasia. Choi et al defined subtypes of non-conventional dysplasia as follows: hypermucinous dysplasia, dysplasia with increased Paneth cell differentiation (DPD), goblet cell-deficient (GCD), crypt cell dysplasia (CCD), sessile serrated lesion (SSL)-like, and traditional serrated adenoma (TSA)-like dysplasia, and serrated lesion NOS [4].

Based on our current knowledge, non-conventional dysplasia may be present in approximately one-fourth to half of patients with IBD-associated colorectal adenocarcinomas [4]. Their importance is highlighted by their tendency to often harbour aneuploidy, to be detected alongside poorly differentiated and signet ring cell carcinomas or flanking advanced neoplasms. Furthermore, hypermucinous, GCD, and CCD subtypes have been associated with flat or even invisible endoscopic morphology. Therefore, their recognition may be challenging and necessitate more extensive and random sampling [5].

We aimed to re-evaluate a cohort of IBD-associated adenocarcinoma cases, retrospectively identify associated non-conventional dysplasias and validate recent North American findings in a Central-Eastern European population. We also provide an updated literature review.

## Materials and methods

A series of 28 randomly chosen cases of known IBD-associated colorectal adenocarcinomas, diagnosed between 2010 and 2022, at the Department of Pathology, University of Szeged was included. In all cases, the patient’s gender, age both at the diagnosis of IBD and neoplasia, type and localisation of IBD, type of specimen [biopsy (n = 8)/surgical specimens (n = 20)], as well as the histological type, grade, localisation, and stage of cancer, disease-free (DFS) and overall survival (OS) were obtained by chart review. Furthermore, all the patients’ prior gastrointestinal histology reports have been reviewed and mean histologic activity of IBD in a 5-year interval before the neoplastic sample was registered. The patients’ index cases yielding the adenocarcinoma diagnosis were independently re-evaluated by two gastrointestinal pathologists (BK, AS), focusing on identifying conventional and non-conventional dysplasias as candidate precursor lesions adjacent to the adenocarcinoma. If present, non-conventional dysplasia was subclassified as hypermucinous, DPD, GCD, CCD, TSA-like, SSL-like, and serrated lesion NOS following the morphologic criteria published by Choi et al [4, 6]. Subsequently, discrepant interpretations were revisited using a multiheaded microscope and discussed to achieve consensus. Statistical analyses were carried out by the R statistical software (v4.1.1). The Mann-Whitney test was used to compare two groups of independent samples (from non-normally distributed data). The association between categorical variables was examined by Fisher’s exact test (with Bonferroni-Holm correction). The Kaplan-Meier method was used to estimate DFS/OS curves, and the logrank test was applied to compare survival curves. All statistical tests were two-sided, and *p*-values of less than 0.05 were considered statistically significant. Kaplan-Meier curves were created using the R package “survminer” (v0.4.9). This study was approved by the institutional ethical committee of the Albert Szent-Györgyi Clinical Centre of the University of Szeged (107/2021-SZTE/RKEB; 4988).

## Results

### Patients’ epidemiological and clinical data

The demographic and clinicopathologic characteristics of the included cases are shown in Table 1. The mean age at carcinoma diagnosis was 47 years in the exclusively conventional and 50 years in the non-conventional dysplastic group. Definite male predominance was found in the examined, IBD-associated adenocarcinoma population in general (male-to-female ratio 22:6). The male-to-female ratios in the exclusively conventional and non-conventional dysplasia groups were 10:1 and 6:2, respectively, which reflects the clear male predominance in both subpopulations. All examined patients were Caucasians.

**TABLE 1:** Epidemiological and clinicopathologic characteristics of the included cohort.

	Conventional dysplasia exclusively (n = 11)	Non-conventional dysplasia exclusively (n = 8)	Mixed conventional and non-conventional cases (n = 9)	*p*-values
Average age (at the time of carcinoma diagnosis)	47	50	49	At last follow-up – *p = 0.218*; at the diagnosis of IBD – *p = 0.275*; at malignant diagnosis –*p = 0.170*
Male:female ratio	10:1	6:2	6:3	*p = 0.524*
Type of IBD	CD = 3; UC = 8	CD = 1; UC = 7	CD = 4; UC = 5	*p = 0.223*
Average duration of IBD	16.1 years (range: 1–50)	15.8 years (range: 0–27)	16.2 years (range: 0–21)	*p = 0.926*
Histological activity of IBD based on prior non-neoplastic biopsies	Active disease (n = 5)	Active disease (n = 6)	Active disease (n = 6)	*p = 1*
Histological subtype of associated colorectal adenocarcinoma	Conventional adenocarcinomas (n = 8), medullary adenocarcinomas (n = 3)	Conventional adenocarcinomas (n = 4), mucinous adenocarcinomas (n = 4)	Conventional adenocarcinomas (n = 9)	*p = 0.014* and *p = 0.041*
Grade of associated colorectal adenocarcinoma	1 (n = 2), 2 (n = 7), 3 (n = 2)	2 (n = 5), 3 (n = 3)	1 (n = 1), 2 (n = 5), 3 (n = 3)	*p = 0.093*
Stage of associated colorectal adenocarcinoma	T1 (n = 1), T3 (n = 7), T4 (n = 3)	T2 (n = 1), T3 (n = 3), T4 (n = 4)	T3 (n = 7), T4 (n = 2)	*p = 0.131*
Location of associated colorectal adenocarcinoma	Left colon (n = 6), right colon (n = 5)	Left colon (n = 3), right colon (n = 5)	Left colon (n = 4), right colon (n = 5)	*p = 0.253*

Abbreviations: CD, Crohn’s disease; IBD, inflammatory bowel disease; NA, not avaliable; UC, ulcerative colitis.

In all groups, the majority of patients suffered from UC. The average duration of IBD at the time of carcinoma diagnosis was 16 years (range: 0–50 years, median: 14). Previous histological samples and reports were not available in 8 cases. Active disease was defined in 12 patients with UC, and 5 CD patients. Active disease was not present in 3 patients. In the exclusively conventional dysplasia group (n = 11), 8 patients were diagnosed with UC, while in the exclusively non-conventional dysplasia group (n = 8), the number of patients was 7, and in the mixed group (n = 9), it proved to be 5. Patients with UC developed adenocarcinoma localised to the colon segment previously reported to be involved by inflammation; in the left (11/20; 55%) and in the right colon (9/20; 45%), while 12.5% (1/8) and 87.5% (7/8) of patients with CD presented with right-sided and left-sided colon cancer, respectively.

From the examined population, only two patients were noted to have primary sclerosing cholangitis (PSC); one belonged to the conventional dysplasia group, while the other had non-conventional dysplasia. Family history was negative for polyposis syndromes in all cases.

**FIGURE 1:**
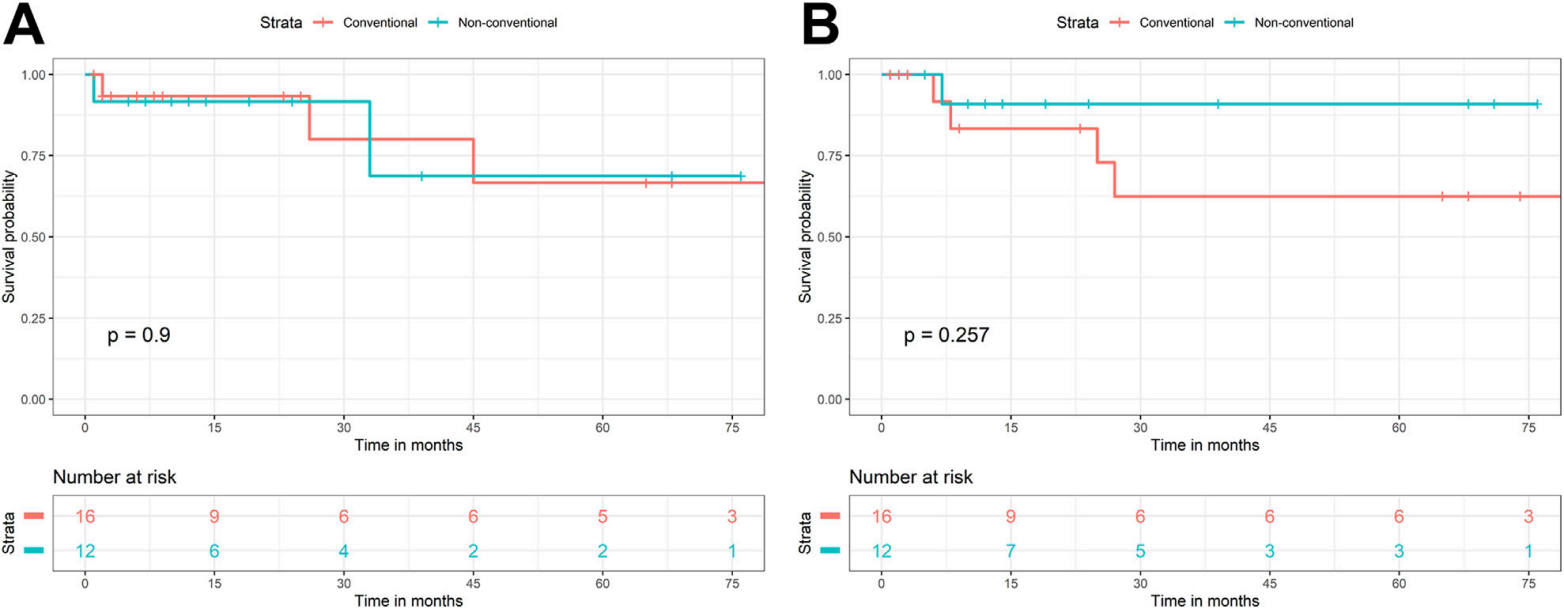
Kaplan-Meier curves of conventional and non-conventional dysplasias, regarding disease-free **(A)** and overall survival **(B)**.

With statistical analysis, there was no significant association found between non-conventional dysplasia and gender (*p = 0.524*), age (at last follow-up – *p = 0.218*; at the diagnosis of IBD – *p = 0.275*; at malignant diagnosis –*p = 0.170*), type of IBD (*p = 0.223*), duration of IBD (*p = 0.926*), and disease activity (*p = 1*). For DFS, the two groups (i.e., patients with conventional and non-conventional dysplasia) had very similar survival curves (*p* = 0.900) (Figure 1A). The differences were also not significant (*p* = 0.257) for the OS (Figure 1B). The Kaplan-Meier curves are displayed in Figure 1.

### Histopathological evaluation and IBD-associated neoplasia

**FIGURE 2:**
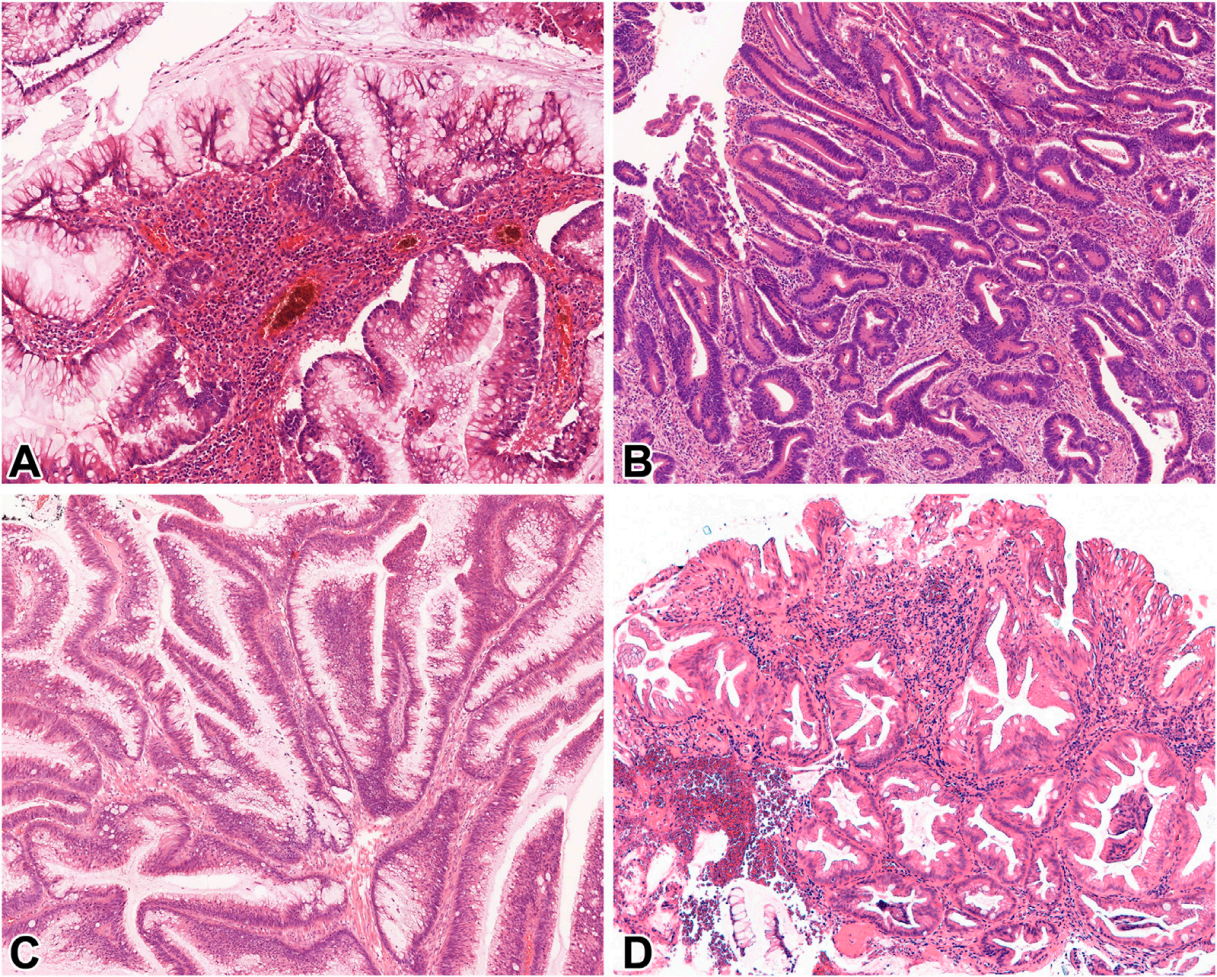
Microscopic features of IBD-associated non-conventional dysplasias of the examined population. **(A)**: Hypermucinous dysplasia (10x, HE), **(B)**: Goblet cell-deficient dysplasia (GCD) (10x, HE), **(C)**: Serrated not otherwise specified (NOS) (10x, HE), **(D)**: Traditional serrated adenoma (TSA)-like dysplasia (10x, HE). Abbreviations: GCD, Goblet cell-deficient dysplasia; HE, Hematoxylin and eosin; NOS, Not otherwise specified; TSA, Traditional serrated adenoma.

Adjacent to the previously reported adenocarcinomas, exclusively conventional dysplasia was detected in 11, while exclusively non-conventional dysplasia in 8 patients. Dysplasia comprised of a combination of conventional and at least one subtype of non-conventional dysplasia was observed in 9 patients. Altogether, 25 non-conventional dysplasia foci were identified, including hypermucinous (n = 9) (Figure 2A), GCD (n = 6) (Figure 2B), serrated lesion NOS (n = 6) (Figure 2C), and TSA-like dysplasia (n = 4) (Figure 2D) subtypes. The co-occurrence of multiple non-conventional dysplasia subtypes within the same case was common (n = 9/17; 53% of all cases with non-conventional dysplasia) in resection specimens. The following combinations were observed: hypermucinous and serrated lesion, NOS (n = 4), hypermucinous and GCD (n = 2), hypermucinous, GCD, and TSA-like (n = 1), GCD and serrated lesion, NOS (n = 1), GCD and TSA-like (n = 1). Half of the 8 cases with only biopsy samples available showed exclusively conventional, while the other half demonstrated exclusively non-conventional dysplasia. None of the included cases in our cohort had a concurrent or prior histologically proven endoscopically non-visible dysplasia in other bowel segments.

Regarding the IBD-associated adenocarcinomas, they were histologically characterised as conventional (n = 8) and medullary (n = 3) in the exclusively conventional, and conventional (n = 4) and mucinous (n = 4) in the exclusively non-conventional dysplasia cases. All mixed cases were associated with conventional adenocarcinomas (n = 9) (Table 1). A significant association (*p = 0.014*) was found between non-conventional dysplasia and adenocarcinoma subtypes, and the proportion of non-conventional dysplasia was significantly different (*p = 0.041*) within mucinous and medullary subtypes. Most examined adenocarcinomas were low-grade (grade 2) in all groups (64% in the exclusively conventional dysplasia, 63% in the exclusively non-conventional dysplasia, and 56% in the mixed group). Regarding stage, most cases were diagnosed as pT3 in the exclusively conventional dysplasia (n = 7) and the mixed group (n = 7), while the adenocarcinomas with exclusively non-conventional dysplasia proved to be mostly T4 (n = 4). Right colon localisation was observed in 5 adenocarcinomas associated with conventional and 5 adenocarcinomas associated with non-conventional dysplasia, while the left colon was affected in 6 cases with adjacent conventional and 3 cases with adjacent non-conventional dysplasia. In the cases associated with mixed dysplasia, left colon localisation was observed in 5, and right colon localisation was seen in 4 patients. No significant association was found between non-conventional dysplasia and the grade (*p = 0.093*), stage (*p = 0.131*), and localisation (*p = 0.253*) of associated adenocarcinomas.

## Discussion with literature review

The literature review was carried out using PubMed search and the keywords “inflammatory bowel disease,” “non-conventional dysplasia,” and “non-conventional dysplasia.”

The classification of IBD-associated dysplasias has changed over the past years. The Riddell classification (negative, indefinite, low-grade, and high-grade) from 1983 has been the gold standard for years; however, new subtypes, including villous and hypermucinous have been introduced in 1999 [7, 8]. The serrated subtype was first identified by Patil et al in 2017 [9]. A more recent and comprehensive classification has been published by Choi et al with 7 non-conventional dysplasia subtypes, including hypermucinous, DPD, GCD, CCD, SSL-like, TSA-like, and serrated lesion NOS [4]. So far, there are 15 original studies in the literature examining these subtypes, of which 8 are retrospective and consecutive, with additional sporadic case presentations [4, 5, 10–23]. Most of the available literature focuses on colorectal pathology; however, counterparts associated with CD affecting the small intestine were also reported [24, 25] Non-conventional dysplasia was detailed in 8 review articles [6, 26–32].

Based on our current knowledge, IBD-associated non-conventional dysplasia may harbour aneuploidy, DNA abnormalities, and p53 mutation (overexpressed or null phenotype) more frequently than conventional counterparts [10, 15]. Regarding their development, high inflammatory activity has been proven to be an independent risk factor, according to the results of Nguyen et al [12]. Musulen et al suggested gastric metaplasia as a candidate precursor lesion to some hypermucinous dysplasia, in accordance with the results of Kővári et al [18, 22]. Furthermore, non-conventional dysplasia in general, is more commonly associated with UC and PSC [6, 11, 15]. In a North American population, DPD and CCD subtypes may be the most common [10]. Moreover, Bahceci et al demonstrated that CCD and GCD frequently present as flat or invisible lesions endoscopically, and most endoscopically undetected lesions were categorised as non-conventional [5, 13].

A correlation between non-conventional dysplasia subtypes and special histologic types of invasive adenocarcinoma was also proposed. GCD and hypermucinous subtypes have been identified as candidate precursor lesions of low-grade tubuloglandular and mucinous adenocarcinomas, respectively [14]. The presence of non-conventional dysplasia has been associated with more frequent and earlier recurrence of intraepithelial neoplasia, larger lesion size, and high-grade adenocarcinomas [4, 16].

Overall, their molecular background, possibly flat or invisible morphology, and the high probability of relapse and high-grade features of associated adenocarcinomas all suggest an overall worse prognosis compared to conventional dysplasia.

In most studies, the evaluation of non-conventional dysplasia has been mainly determined by one or two expert gastrointestinal pathologists [5, 10–15]; therefore, the reproducibility of the new classification is uncertain. Although each included 6 evaluators, Choi et al’s and Lang-Schwartz et al’s studies did not report data on intraclass correlation. According to the results of Nasreddin et al, poor agreement was found between 6 evaluators using the same classification [17].

An even more detailed classification was published in 2023 by Harpaz et al, dividing IBD-associated dysplasias into intestinal (tubular/villous adenoma-like, GCD, CCD, TSA-like, SSL-like, serrated NOS), gastric (tubular/villous, serrated), and mixed categories. Reproducibility examination reflected good general agreement regarding definitive diagnosis [19]. Table 2 summarises the results of the literature review.

**TABLE 2:** Results of the literature review.

Author	Year of publication	Type of article	Type of study	Examined subtypes	Number of cases	Number of evaluators	Types of specimens	Immunohistochemical analysis	Genetic analysis	New findings
Andersen et al. [8]	1999	Original research	Retrospective, consecutive, unicenter	Hypermucinous, villous mucosa	13	Not mentioned	Colectomy	NA	KRAS mutation analysis	Highest KRAS mutation frequency was found in hypermucinous and villous mucosa
Patil et al. [9]	2017	Original research	Retrospective	New classification: adenoma-like, terminally differentiated, serrated, hypermucinous	30	Not mentioned	Not mentioned	MUC2, MUC5AC, MUC6, p53, β-catenin, annexin A10, Maspin, BRAF, SOX9	NA	P53 plays a role in adenoma-like, terminally differentiated, and hypermucinous dysplasias. Combined alterations of p53 and β-catenin are observed in serrated precursors. MUC6 is a marker of hypermucinous subtype
Kamarádová et al. [33]	2019	Original research	Retrospective, consecutive, unicenter	Putative precursor lesions (Serrated change/dysplasia, villous hypermucinous change)	309	Not mentioned	Colectomy	MMR, p53, MGMT	KRAS, NRAS, BRAF mutation analysis	IBD-associated adenocarcinomas are heterogenic. All putative precursor lesions are associated with longstanding IBD
Kamarádová et al. [34]	2020	Original research	Retrospective, consecutive, unicenter	Putative precursor lesions (Serrated change/dysplasia, villous hypermucinous change)	309	Not mentioned	Colectomy	MLH1, p53, MGMT	KRAS, NRAS, BRAF mutation analysis	Almost half of IBD-associated non-conventional dysplasias may harbour KRAS mutations
Choi et al. [4]	2020	Original research	Retrospective, multicenter	New classification: Hypermucinous, TSA-like, SSL-like, serrated NOS, DPD, GCD, CCD	58	6	Colectomy	NA	NA	Colorectal carcinomas associated with non-conventional dysplasias tend to be high-grade, and they mainly be found in the left colon
Lee et al. [10]	2021	Original research	Retrospective, consecutive, unicenter	Hypermucinous, TSA-like, SSL-like, serrated NOS, DPD, GCD, CCD	168	1 or 2	Biopsy, colectomy	NA	DNA flow cytometry	Almost half of non-conventional dysplasias may harbour aneuploidy, and they may present as flat lesions during endoscopic examination. DPD and CCD are the most commonly identified subtypes
Choi et al. [5]	2022	Original research	Retrospective, multicenter	Hypermucinous, CCD, GCD	126	1	Biopsy, colectomy	p53	NA	Non-conventional dysplasias may predominantly be found in patients with UC, and may be associated with PSC in 1/4 of cases. CCD and GCD subtypes are often present as endoscopically flat or invisible lesions. P53 null or mutant phenotype may be found in 29%–55% of cases
Zhang et al. [11]	2022	Original research	Retrospective, consecutive, unicenter	Hypermucinous, TSA-like, SSL-like, serrated NOS, DPD, GCD, CCD	173	2	Biopsy, colectomy	NA	NA	Almost 1/3 of PSC-IBD patients may develop dysplasia, that is mostly characterized as non-conventional
Nguyen et al. [12]	2022	Original research	Retrospective, consecutive, unicenter	Hypermucinous, TSA-like, SSL-like, serrated NOS, DPD, GCD, CCD	125	1	Biopsy	NA	NA	Higher inflammatory activity score increases the chance of the development of IBD-associated non-conventional dysplasias
Bahceci et al. [13]	2022	Original research	Retrospective, consecutive, unicenter	Hypermucinous, TSA-like, SSL-like, serrated NOS, DPD, GCD, CCD	207	2	Biopsy, colectomy	NA	NA	Colonoscopically undetected dysplasias are mainly non-conventional
Akarca et al. [14]	2023	Original research	Retrospective, consecutive, multicenter	Hypermucinous, TSA-like, SSL-like, serrated NOS, DPD, GCD, CCD	46	1	Colectomy	NA	NA	CCD and hypermucinous dysplasias are precursor lesions of low-grade tubuloglandular and mucinous adenocarcinomas
Zhang et al. [15]	2023	Original research	Retrospective, consecutive, unicenter	Hypermucinous, TSA-like, SSL-like, serrated NOS, DPD, GCD, CCD	96	1	Biopsy	NA	DNA flow cytometry	PSC-IBD patients are more likely to developing dysplasias of the right colon, and DNA abnormality
Lang-Schwartz et al. [16]	2023	Original research	Retro- and prospective, consecutive, unicenter	Hypermucinous, DPD, GCD, CCD	5435	6	Not mentioned	NA	NA	Non-conventional dysplasias are associated with more frequent and earlier relapse low-grade intraepithelial neoplasia relapse, and larger lesion size
Harpaz et al. [19]	2023	Original research	Retrospective	New classification: intestinal dysplasia (tubular/villous adenoma-like, GCD, CCD, TSA-like, SSL-like, serrated NOS), gastric dysplasia (tubular/villous, serrated), and mixed	35	7	Not mentioned	NA	NA	The new classification system is reproducible, with 2/3 of cases with definitive diagnosis
Nasreddin et al. [17]	2024	Original research	Retrospective, consecutive, unicenter	Hypermucinous, TSA-like, SSL-like, serrated NOS, DPD, GCD, CCD	89	6	Biopsy, colectomy	NA	NA	Poor agreement IBD-associated was found in IBD-associated non-conventional dysplasia subtypes
Musulen et al. [18]	2024	Original research	Retrospective, multicenter	Hypermucinous, TSA-like, SSL-like, serrated NOS, DPD, GCD, CCD, serrated epithelial change	33	3	Colectomy	p53, MUC5AC, MLH1, CDX2		On the basis of IBD, gastric metaplasia may develop, that serves as a precursor lesion of non-conventional dyplasias. The expression of MUC5AC decreases with the increase of the degree of dysplasia
Our study	2024	Original research	Retrospective, randomized	Hypermucinous, TSA-like, SSL-like, serrated NOS, DPD, GCD, CCD	28	2	Biopsy, colectomy	NA	NA	IBD-associated non-conventional dyplasias may often appear combined, and the most common combination is hypermucinous and serrated NOS. Non-conventional dysplasias are significantly associated with mucinous adenocarcinomas, while conventional subtypes areassociated with medullary adenocarcinomas

Abbreviations: BRAF, v-Raf murine sarcoma viral oncogene homolog B; CD, Crohn’s disease; CCD, Crypt cell dysplasia; CDX2, Caudal-type homeobox transcription factor 2; CK7, Cytokeratin 7, CK20, Cytokeratin 20; DNA, Deoxyribonucleic acid; DPD, Dysplasia with increased Paneth cell differentiation; GCD, Goblet cell-deficient dysplasia; IBD, Inflammatory bowel disease; IDH1, Isocitrate dehydrogenase 1; KRAS, Kirsten rat sarcoma virus; Maspin, Mammary serine protease inhibitor; MGMT, O (6)-Methylguanine-DNA-methyltransferase; MLH1, MutL homolog 1; MMR, Mismatch repair; MSH2, MutS homolog 2; MSH6, MutS homolog 6; MUC2, Mucin 2; MUC5AC, Mucin-5AC; MUC6, Mucin 6; NA, Not available; NRAS, Neuroblastoma ras viral oncogene homolog; NOS, Not otherwise specified; p53, Tumour protein p53; PMS2, PMS1 homolog 2; PSC, Primary sclerosing cholangitis; SOX9, SRY-box transcription factor 9; SSL, Sessile serrated lesion; TSA, Traditional serrated adenoma; UC, Ulcerative colitis.

Hereby, we performed the first Hungarian pilot study, aiming to re-evaluate several IBD-associated adenocarcinoma cases to retrospectively survey the incidence of non-conventional dysplasia and to validate that recent North American findings may apply to a Central-Eastern European population. We also provide an updated literature review.

Our study successfully demonstrated the presence of non-conventional dysplasia adjacent to 60% of 28 randomly selected IBD-associated colorectal adenocarcinoma cases diagnosed between 2010 and 2022 at the Department of Pathology, University of Szeged. Although our primary focus was identifying and subtyping adjacent non-conventional dysplasias, we also aimed to evaluate the most significant clinicopathological parameters. In our study, the most common subtype proved to be hypermucinous dysplasia, but interestingly, CCD and DPD subtypes were not found in the examined population, even though these subtypes are common in North American populations [4–6]. Nonetheless, DPD was reported to be typically low-grade and less frequently associated with advanced neoplasms, explaining its absence in our IBD-associated adenocarcinoma cohort [6]. Otherwise, discrepancies may be explained by the differing population or the non-consecutive and pilot nature of our study design. Some examined clinicopathological parameters, including patients’ age at the time of dysplasia diagnosis, the duration of IBD, and the association with UC reflect international literature data. Other prognostic factors of colorectal carcinomas showed varying distribution, likely due to the low number of cases; therefore, no significant association was found between these parameters. Although literature data supports an association between the risk of non-conventional dysplasia development and high histologic inflammatory activity, we failed to reproduce these findings, likely due to the lack of statistical power [12]. A significant association was found between adenocarcinoma subtypes and adjacent dysplasia subtypes. Mucinous adenocarcinomas were solely found in the non-conventional dysplasia population, and medullary adenocarcinomas were only present in the conventional dysplasia group. Unexpectedly, all cases with mixed conventional and non-conventional dysplasia components were associated with conventional adenocarcinomas (n = 9). This finding is discrepant from the results of earlier studies, which showed mixed dysplasia associated with both tubuloglandular and mucinous adenocarcinomas [14]. The recent studies of Kővári et al and Musulen et al may offer some explanation for these results, as some evidence suggests that a chronic inflammation-foveolar metaplasia-hypermucinous dysplasia-mucinous adenocarcinoma sequence may exist in the setting of IBD [18, 22]. The association of non-conventional dysplasias with UC was reproduced in our cohort.

The limitation of our study, as expected from a pilot design, is the relatively small number of patients. We are currently working on a complete consecutive IBD-associated neoplasia cohort comprising all cases between 2010 and 2023. The selection of cases was random, and unexpectedly, all cases included dysplasia adjacent to the carcinoma. Based on our results, either conventional, non-conventional or mixed, dysplasia is a common tumour-accompanying change in the setting of IBD. Although non-conventional dysplasias were reported to harbour molecular alterations characteristic of advanced neoplasia [5, 10], more detailed comparative molecular analysis of both the carcinomatous and dysplastic components are needed to establish that non-conventional dysplasias represent definite tumour precursor lesions. Of note, none of the included cases in our cohort had concurrent or prior histologically proven, endoscopically non-visible dysplasia. However, the retrospective nature of this study may explain the lack of such lesions. At the time of sampling, endoscopists were unaware of the implications of non-conventional IBD-related dysplasia and its association with flat and invisible endoscopic presentation. In most of our cases with prior or concurrent biopsies targeting other colonic segments, the samples were taken from endoscopically inflamed mucosa to determine disease activity, endoscopically visible lesions concerning for dysplasia, or represented limited step biopsies from endoscopically uninvolved mucosa. A further limitation is the lack of insight into the clinical management (i.e., the type of anti-inflammatory drug used for induction and maintenance of remission and the applied dosages), that may have had an impact on the ongoing inflammation-related mucosal injury and related carcinogenetic damage.

International data and our results highlight the importance of recognising IBD-associated non-conventional dysplasias on the pathologists’ side. At the same time, clinically, these patients may benefit from individualised follow-up and random biopsy sampling.

## Data Availability

The datasets presented in this article are not readily available because the dataset was built from the IBD patients treated at the University of Szeged, and will be later used for further studies. Requests to access the datasets should be directed to AS, sejben.anita@gmail.com.

## Glossary

AJCC: American Joint Committee on Cancer
BRAF: v-Raf murine sarcoma viral oncogene homolog B
CCD: Crypt cell dysplasia
CD: Crohn’s disease
CDX2: Caudal-type homeobox transcription factor 2
CK7: Cytokeratin 7
CK20: Cytokeratin 20
CRC: Colorectal cancer
DFS: Disease-free survival
DNA: Deoxyribonucleic acid
DPD: Dysplasia with increased Paneth cell differentiation
GCD: Goblet cell-deficient dysplasia
HE: Hematoxylin and eosin
IBD: Inflammatory bowel disease
IDH1: Isocitrate dehydrogenase 1
IMP3: Insulin-like growth factor II messenger ribonucleic acid binding protein 3
KRAS: Kirsten rat sarcoma virus
LGTGA: Low-grade tubuloglandular adenocarcinoma
MAC: Mucinous adenocarcinoma
MASPIN: Mammary serine protease inhibitor
MGMT: O(6)-Methylguanine-DNA-methyltransferase
MLH1: MutL homolog 1
MMR: Mismatch repair
MSH2: MutS homolog 2
MSH6: MutS homolog 6
MSI: Microsatellite instability
mRNA: Messenger ribonucleic acid
MUC2: Mucin 2
MUC5AC: Mucin-5AC
MUC6: Mucin 6
NA: Not available
NOS: Not otherwise specified
NRAS: Neuroblastoma ras viral oncogene homolog
OS: Overall survival
PIK3CA: Phosphatidylinositol-4,5-bisphosphate 3-kinase catalytic subunit alpha
PMS2: PMS1 homolog 2
p53: Tumour protein p53
PSC: Primary sclerosing cholangitis
SOX9: SRY-box transcription factor 9
SSL: Sessile serrated lesion
TNM: Tumour, Node, Metastasis
TSA: Traditional serrated adenoma
UC: Ulcerative colitis
US: United States
